# Idiopathic Intracoronal Resorption of Permanent Molars: A Report of Two Cases

**DOI:** 10.1155/crid/9610040

**Published:** 2025-02-22

**Authors:** Krasimir Hristov, Victoria Zlateva

**Affiliations:** Department of Pediatric Dentistry, Faculty of Dental Medicine, Medical University of Sofia, Sofia, Bulgaria

**Keywords:** incomplete root development, intracoronal resorption, vital pulp therapy

## Abstract

Intracoronal resorption (IR) is a condition characterized by the presence of lesions in the dentin of unerupted or erupting teeth, usually located just below the enamel–dentin junction in the occlusal part of the crown. This article presents two cases of IR—one with and one without pulp involvement. In both cases, the teeth were asymptomatic, and the lesions were discovered during routine checkups. The first case presents pre-eruptive IR with pulp involvement in an immature mandibular molar. To preserve the vitality of the growth zone and complete root development, vital pulp therapy was performed through total coronal pulpotomy and application of Biodentine. The second case is also of a mandibular molar with incomplete root development, but although it initially resembled invasive cervical resorption, treatment revealed that the granulation tissue was surrounded by intact enamel and did not affect the pulp or root of the tooth. No carious lesion was found. The tooth was conventionally restored with a composite filling.

## 1. Introduction

Resorption is a condition associated with a physiological or pathological process leading to the loss of dentin, cementum, or alveolar bone [1]. While resorption of primary teeth is an example of physiological resorption without a microbial component, in permanent teeth, resorption is pathological and can be broadly classified as external and internal [2].

Internal resorption begins in the pulp and involves the resorption of surrounding dentin, with various factors such as trauma and orthodontic treatment being discussed as potential causes [3–5]. Treatment options depend on clinical findings and may include monitoring, root canal treatment, or extraction [3].

External cervical resorption involves the loss of hard dental tissues in the cervical area of the tooth, with factors such as trauma and dental procedures suggested as potential causes [6]. Treatment approaches vary and may include nonsurgical access, flap opening, or endodontic access [7].

Intracoronal resorption of unerupted teeth, often idiopathic, most commonly affects permanent molars and premolars [8]. Although some cases may result from anomalies during crown development, most are considered to be caused by external resorption [9]. Recent data suggest that resorptive cells, including osteoclasts and chronic inflammatory cells, penetrate the dentin through microperforations in the crown. This process occurs before enamel maturation or near the cementoenamel junction, with resorptive cells originating from the surrounding bone or the developing dental follicle [10]. Their frequency, according to some studies, varies between 0.5% and 2%, and according to others, 0.2% and 3.14%, regardless of gender, race, systemic factors, and the patient's general medical status [11–13].

It is most commonly found in mixed dentition (7–10 years), affecting the occlusal surface in 87% of cases and typically reaching up to two-thirds of the dentin thickness. Only about 9% of the lesions show a tendency to progress within 3 years [10].

Previous studies found that pre-eruptive intracoronal resorption (PEIR) affects only the crown of the tooth, starting at the enamel–dentin junction and extending to varying depths in the dentin [14–16]. Recent studies have found cases where the resorption also affects the root of the tooth. This led to the development of a new classification system in 2022, which provides the clearest description of resorption lesions by encompassing the enamel, dentin, pulp, and root of the tooth. Using this system, all lesions are recorded with codes from 1 to 15 [17].

A possible initiating factor for such intracoronal resorption is the destruction of the reduced enamel epithelium, which can lead to the invasion of resorptive connective tissue into the dentin [18]. Ectopic positioning of the tooth can also act as a triggering factor due to the creation of local pressure that stimulates the resorption process [12]. In such cases, local or systemic etiological factors cannot be identified, although the lesions may be triggered by the same predisposing factors as external cervical resorption [19]. Diagnosing this condition can be done before or after tooth eruption, with most cases lacking accompanying clinical symptoms, making diagnosis difficult [12]. A recent systematic review highlights that surgical opening and restoration are the top treatment choices for PEIR, representing 36% of cases. Treatment plans are also customized based on the lesion's progression and the tooth's eruption state to ensure the most effective approach for each patient [20].

This article presents two clinical cases of intracoronal resorption of erupting molars—one with and one without pulp involvement—highlighting the importance of early diagnosis and appropriate management. Consent to publish was obtained from both patients and their parents before proceeding.

## 2. Case Reports

### 2.1. Clinical Case 1

A 7.5-year-old girl was referred to the Faculty of Dental Medicine in Sofia by her orthodontist, who noticed an unusual pinkish shadow on the erupting Tooth 36. The girl was systemically healthy and not on any medication. No endogenous fluoride prophylaxis has been conducted. Extraoral examination revealed no significant findings.

**Figure 1:**
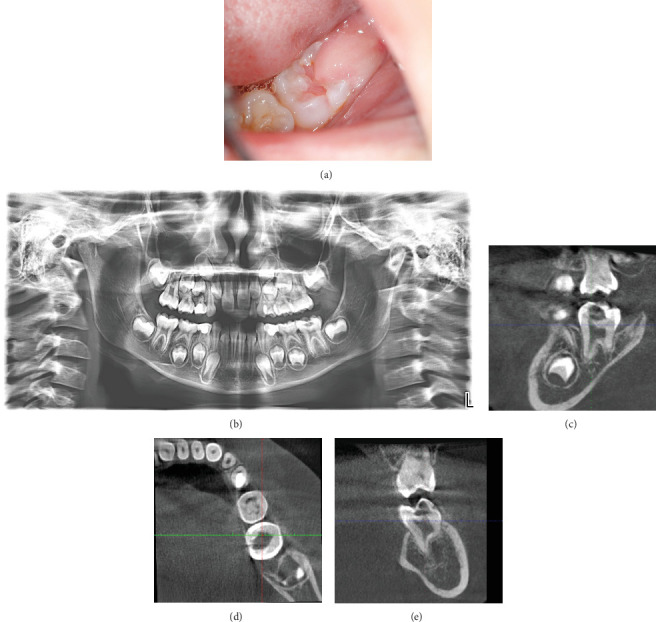
(a) Intraoral preoperative image; (b) panoramic X-ray taken 5 months earlier for orthodontic indications; (c) CBCT—sagittal view; (d) CBCT—transverse view; and (e) CBCT—coronal view.

During the clinical intraoral examination, a partially erupted Tooth 36 was observed with an operculum covering the distal half of the occlusal surface. There was a noticeable enamel cavitation with pink, painful upon probing soft tissue in the central fissure area. A pink shadow was visible under the enamel (Figure 1(a)). The tooth responded normally to cold testing (Pulp Spray, Cerkamed, Stalowa Wola, Poland)—pain appeared immediately and disappeared right after the stimulus was removed. There was no pain on pressure or percussion, and no pathological mobility.

A panoramic X-ray from 5 months earlier showed a round, faint shadow occlusally and incomplete root development with formed root walls and unformed apex (Figure 1(b)). To determine the extent of the lesion, a cone beam computed tomography (CBCT) scan with a field of view (FOV) of 8 × 8 cm was utilized to capture detailed images of the affected area. Involvement of the pulp was observed in the region of the mesiobuccal pulp horn (Figures 1(c), 1(d), and 1(e)). After explaining the procedure and its associated risks, informed consent was obtained from both the child and her parents.

**Figure 2:**
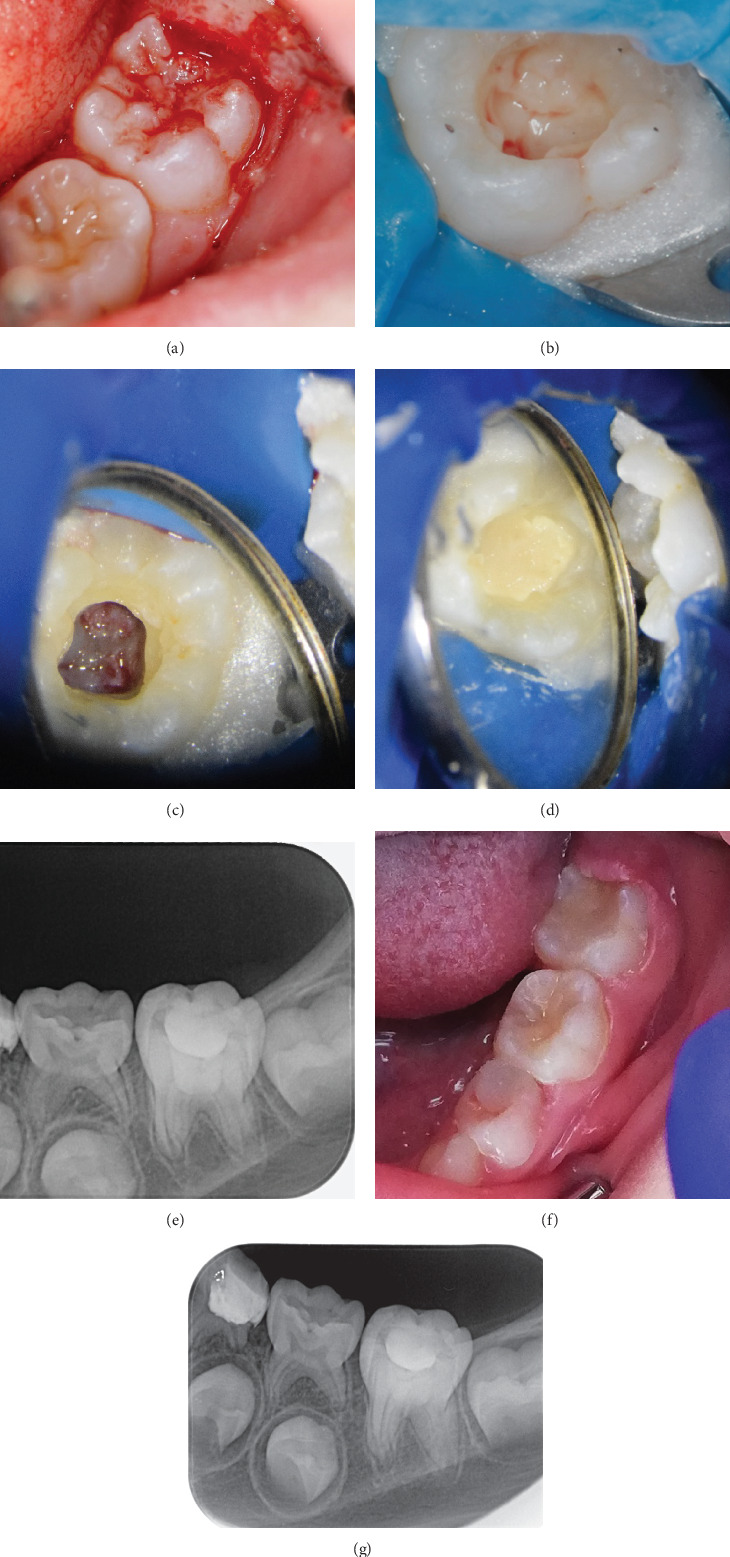
(a) Intraoral image after gingivectomy; (b) pulp exposure in the mesiobuccal pulp horn; (c) image after coronal pulpotomy and hemostasis; (d) applied Biodentine and glass ionomer cement; (e) postoperative control radiograph; (f) condition of the soft tissue after 1 week; and (g) 6 months follow-up.

An inferior alveolar nerve block with Dentocaine 40/0.005 mg/mL (Articaine hydrochloride 40 mg/mL and epinephrine 0.005 mg/mL) was administered. The operculum was excised with a Therma-Cut bur (Dentsply Sirona; York, USA) (Figure 2(a)). A winged molar clamp (#14) (Coltene; Altstätten, Switzerland) and NicTone sheets (Nic Tone; Mexico City, Mexico) were used for rubber dam placement, and the lesion was exposed occlusally with a diamond bur under water cooling. The exposed soft tissue was removed, reaching the exposure site in the area of the mesiobuccal pulp horn, as diagnosed on CBCT (Figure 2(b)).

Given the extent of the exposure, which exceeded 1 mm, partial pulpotomy was initiated. After removing 2 mm of the pulp, two separate 5-min attempts were made to control the bleeding, both of which were unsuccessful. Due to the incomplete root development, the next most conservative treatment option was a coronal pulpotomy performed with a new sterile diamond bur to the level of the orifices to preserve vital pulp in the root canals and growth zone, ensuring root development completion. Pulp bleeding was slight and bright red. Irrigation with 2% sodium hypochlorite and 0.9% saline solution was performed, followed by hemostasis with gentle pressure using a sterile cotton pellet for 2–3 min (Figure 2(c)). Biodentine (Septodont Ltd., Saint Maur des Faussés, France) was applied as a pulp capping agent, followed by a base of glass ionomer cement (GC Fuji TRIAGE, GC Corporation, Tokyo, Japan; Figure 2(d)) and composite restoration (VisCalor bulk, VOCO GmbH, Cuxhaven, Germany).

Postoperative X-ray was performed (Figure 2(e)), and clinical examination was scheduled for 1 week later (Figure 2(f)). Follow-ups were also scheduled for the third and sixth months, the first year, and once a year for 5 years. A postoperative radiograph taken 6 months after the procedure showed ongoing root development (Figure 2(g)).

### 2.2. Clinical Case 2

A 12-year-old girl was referred to the Faculty of Dental Medicine. The girl was systemically healthy and not on any medication. She had no subjective symptoms. Extraoral examination revealed no significant findings.

**Figure 3:**
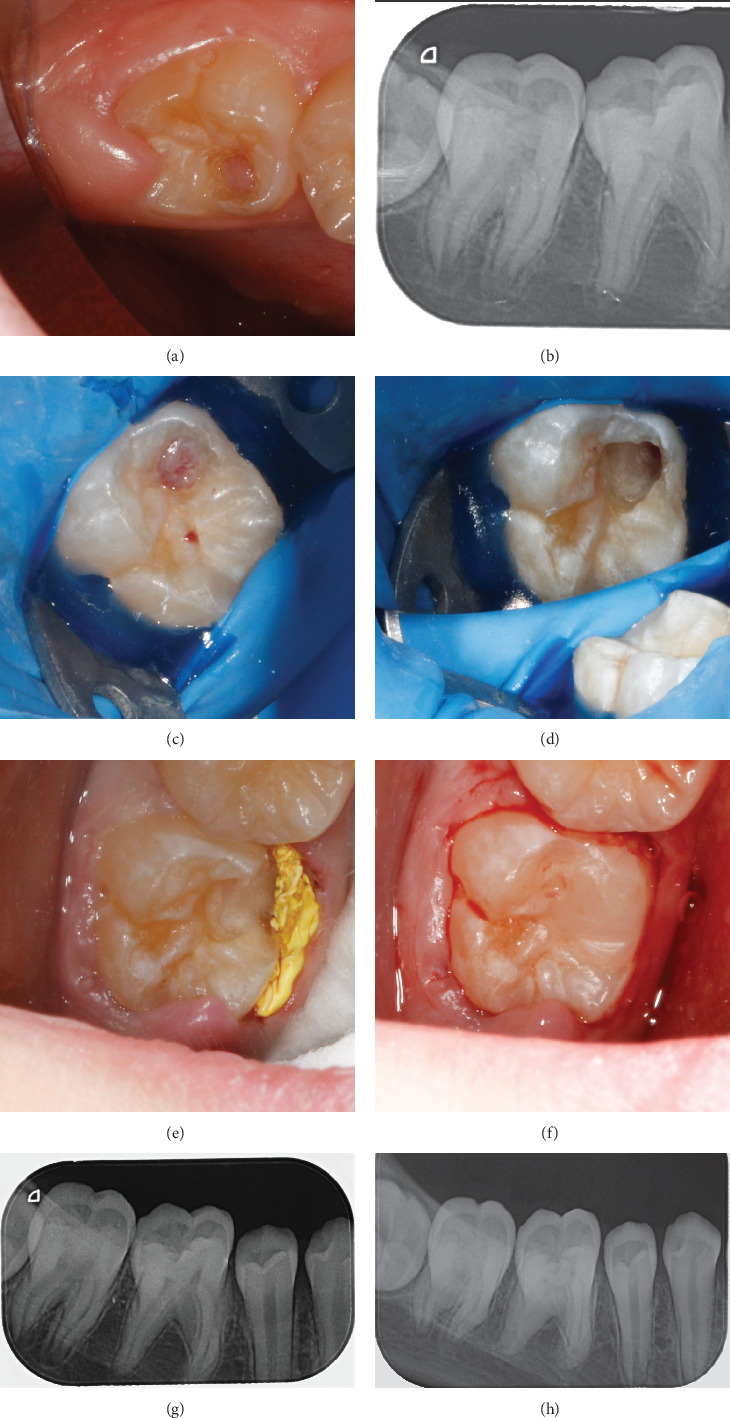
(a) Preoperative image of tooth; (b) preoperative radiograph; (c) isolation with a rubber dam; (d) after exposing the lesion and removing the existing soft tissue; (e) gingival retraction with Teflon tape; (f) composite restoration; (g) postoperative radiograph; (g) postoperative radiograph; and (h) 6 months follow-up.

During the clinical examination, a partially erupted Tooth 47 was observed with the gingiva covering a small part of the distal occlusal surface. In the mesiolingual cusp area, cavitation with prominent soft tissue, painful upon probing, was noted (Figure 3(a)). The tooth responded normally to cold testing (Pulp Spray, Cerkamed, Stalowa Wola, Poland). There was no pain on pressure or percussion, and no pathological mobility. A preoperative X-ray revealed a tooth with incomplete root development with formed root walls and unformed apex. There were no radiographic signs of pulp involvement (Figure 3(b)). A CBCT was recommended, but the parents declined due to concerns about high radiation exposure. After outlining the procedure and its associated risks, we obtained informed consent from both the child and her parents.

An inferior alveolar nerve block with Dentocaine 40/0.005 mg/mL (Articaine hydrochloride 40 mg/mL and epinephrine 0.005 mg/mL) was administered, and the tooth was isolated with a rubber dam (Figure 3(c)). The lesion was exposed with a diamond bur under water cooling. It was found that the entrance to the lesion was subgingival, on the lingual wall, surrounded by enamel without affecting the root or pulp (Figure 3(d)). Due to the inability to ensure adequate isolation, the rubber dam was removed, and gingival retraction was performed with a Teflon tape (Figure 3(e)). This was followed by adhesive restoration with a light-curing composite (G-aenial A'chrod, GC Corporation, Tokyo, Japan; Figure 3(f)).

A clinical examination was scheduled for 1 week later. Six months posttreatment, a follow-up X-ray revealed continued root development.

## 3. Discussion

The presented clinical cases describe intracoronal resorption in erupting permanent molars with and without pulp involvement. This shows that intracoronal resorption can vary in presentation among different patients, which aligns with cases described in the literature [12, 18, 21, 22]. It is important to assess the rate of progression, as it is crucial in choosing the treatment method. In cooperative patients with low caries risk and slow-progressing lesions, the use of sealants without operative treatment is a possible approach [8]. Due to pulp involvement in one of the presented cases and the risk of pulp complications in the other, the operative approach was deemed the most appropriate for treating the presented cases.

For a resorptive lesion to progress, the pulp tissue apical to the resorption must be vital with a preserved blood supply. If treatment is not conducted, this tissue will necrotize, and the entire root canal system will become infected, leading to the development of apical periodontitis [23]. Delayed diagnosis of a tooth with pre-eruptive resorption may lead to the development of an acute periapical abscess and the need for root canal treatment of the affected teeth. This emphasizes the importance of early diagnosis through radiographs to minimize the destructive capacity of the resorptive process [7].

Clinical Case 1 demonstrates the treatment of PEIR in a tooth with incomplete root development involving the pulp. In such cases, it is extremely important to preserve the vital growth zone to ensure root development. Due to the risk of possible infection and inflammation of the pulp in the area of exposure, as well as the lack of information about the nature of the resorptive tissue, and the presence of uncontrollable bleeding, total coronal pulpotomy and pulp capping with a bioactive material were chosen as the treatment method to stimulate the healing process and complete root development [24].

In Clinical Case 2, the lesion resembled invasive cervical resorption. During treatment, it was found that the entrance to the lesion in the crown was surrounded by enamel without affecting the root or pulp. Therefore, this type of lesion is different from internal or invasive cervical root resorption. In both presented cases, mandibular molars in girls were affected. This coincides with recent studies proving that intracoronal resorption is more common in girls than in boys and affects mandibular molars six times more often than maxillary molars. The mechanism and reasons for this are unknown [17, 25].

The presented clinical cases pose a significant challenge due to the unclear etiology and the lack of established protocols for treatment. Some limitations should be mentioned, most importantly the absence of histological examination to confirm the nature of the examined tissue and the short 6-month follow-up period.

## 4. Conclusion

Pediatric dentists should be familiar with the different types of external and internal resorption, as it can be diagnosed in childhood. Early diagnosis and the correct treatment approach according to the clinical case are key to treating these lesions. Special attention should be paid to resorption in teeth with incomplete root development, given the need to select the right material and technique to create conditions for preserving the radicular pulp and completing root development.

## Data Availability

The data that support the findings of this study are available from the corresponding author upon reasonable request.
